# Odour boosts visual object approach in flies

**DOI:** 10.1098/rsbl.2020.0770

**Published:** 2021-03-17

**Authors:** Karen Y. Cheng, Mark A. Frye

**Affiliations:** UCLA Department of Integrative Biology and Physiology, Los Angeles, CA, USA

**Keywords:** feature detection, olfaction, multisensory, insect flight, *Drosophila*

## Abstract

Multisensory integration is synergistic—input from one sensory modality might modulate the behavioural response to another. Work in flies has shown that a small visual object presented in the periphery elicits innate aversive steering responses in flight, likely representing an approaching threat. Object aversion is switched to approach when paired with a plume of food odour. The ‘open-loop’ design of prior work facilitated the observation of changing valence. How does odour influence visual object responses when an animal has naturally active control over its visual experience? In this study, we use closed-loop feedback conditions, in which a fly's steering effort is coupled to the angular velocity of the visual stimulus, to confirm that flies steer toward or ‘fixate’ a long vertical stripe on the visual midline. They tend either to steer away from or ‘antifixate’ a small object or to disengage active visual control, which manifests as uncontrolled object ‘spinning’ within this experimental paradigm. Adding a plume of apple cider vinegar decreases the probability of both antifixation and spinning, while increasing the probability of frontal fixation for objects of any size, including a normally typically aversive small object.

## 1. Introduction

In flight, flies approach the vertically elongated edges of landscape features such as plant stalks, whereas they avoid threats posed by small moving objects [1–3]. This simple algorithm, based only on vertical object size, reduces the computational resources required for the brain to quickly make a crucial behavioural decision [1]. In free-flight, this behavioural decision happens during a single turn—within a fraction of a second—but the valence of a visual feature has been shown to persist far longer [2]. Under so-called ‘open-loop’ experimental conditions, in which the wing kinematics of a tethered fly are recorded in response to imposed visual stimuli but the animal cannot control its visual experience, flies steer towards a tall object projected into the visual periphery and away from a small object in the same location for seconds [1,2,4], an artificially elongated time frame. When provided with virtual ‘closed-loop’ feedback, in which the fly's steering effort controls the visual stimulus [1,5], persistent approach towards a bar manifests as centring the object on the visual midline. Under closed-loop control, object aversion manifests either as spinning, in which a fly seems to forego active control and instead steers constantly in one direction, or as antifixation, in which a fly actively avoids the stimulus, keeping it centred in the rear field of view [1].

For *Drosophila melanogaster*, the presentation of an attractive odour modulates the attractiveness of small objects [2,6]. Mechanistically, under open-loop tethered flight in which a peripheral object evokes tonic aversion, odour switches the steering valence from avoidance to approach [1,4]. However, under natural flight conditions, object position would vary with steering effort. How does food odour modulate visual object valence when the animal has active control over the trajectory of the object?

We sought to answer this question using a standard virtual closed-loop flight simulator. We compared how flies actively control the spatial location of three visual objects in odourless air and in a plume of the naturally appetitive odour apple cider vinegar [7]. We measured the influence of odour on three visual control modes: fixation, spinning and antifixation. We confirm that for progressively taller objects flies show less antifixation, less spinning, and more fixation. We then show that odour further decreases both antifixation and spinning, while increasing frontal fixation of all objects.

## 2. Methods

**Figure 1.**
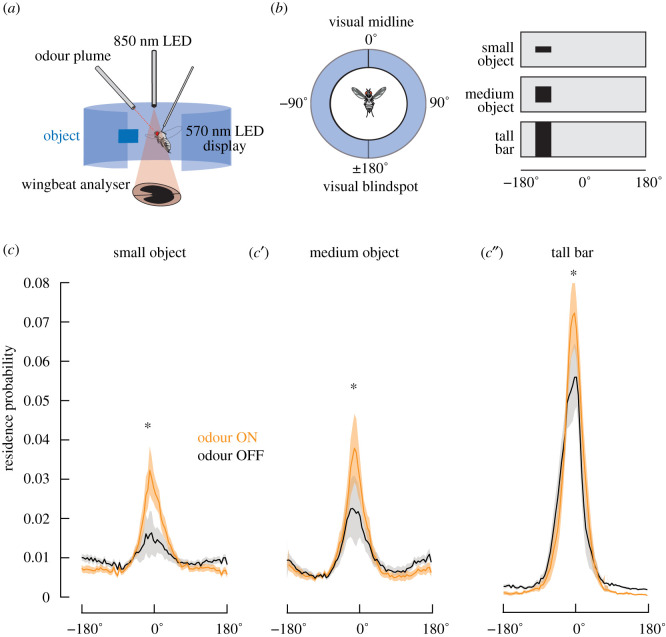
(*a*) Schematic of the apparatus. A tethered fly is suspended within a cylinder of LED panels. Odour vapour is delivered from a nozzle. An 850 nm LED supplies a wingbeat analyser measuring steering effort to control the angular velocity of the visual stimulus object. (*b*) (Left) The visual display from above; 0° is visual midline. (Right) Visual stimuli represented on an unwrapped, linear display. (*c–c*″) Azimuthal residency probability for air (black) and odour (orange). Solid lines represent the mean (*n* = 17), shaded regions ± s.e.m. *p < 0.05, Student's paired *t*-test.

Three- to five-day-old female wild-type flies (*D. melanogaster*) reared from an iso-female line were used [8]. Flies were removed from food, rigidly tethered at the dorsal thorax (the head was not immobilized) onto a 0.1 mm-diameter tungsten pin and allowed to rest for 1 h. A tethered fly was suspended in the centre of a circular display of 570 nm light emitting diodes (LEDs) [9] with a separate infrared wingbeat analyser to record wingbeat amplitude and frequency (figure 1*a*). The steering effort, proxied as the difference between left and right wingbeat amplitudes, ΔWBA [10], was negatively coupled to the angular velocity of the visual stimulus such that when the fly steered in one direction, the visual stimulus moved in the opposite direction to ‘virtually’ close the control loop. A mass-flow-regulated odour plume (40 ml min^−1^) was delivered through a 20 µl pipette tip suspended 1 cm fronto-dorsal of the fly's head [2,11] (figure 1*a*). Apple cider vinegar (Ralph's Grocery generic brand) diluted 70% in water was interspersed with water vapour in a randomized fashion.

Visual stimuli were composed of solid dark objects set against a bright equiluminant background, sized 7.5° × 30° ‘small object’, 30° × 30° ‘medium object’ and 94° × 30° ‘tall bar’ (figure 1*b*, right). Visual objects were presented randomly, appearing behind the fly at 180° for each trial. The 20 s trials were repeated six times per odour condition at a closed loop gain of −20 frames s^−1^ per volt of ΔWBA. Trials were interspersed 8 s periods of closed-loop with a 94° × 15° bar at −10 frames s^−1^ gain. Experiments generally lasted 5 h. All control, acquisition and analysis was performed with custom Matlab scripts.

Analysis was similar to that used previously [1]. Stimulus position was sampled at 1 kHz from flies whose wingbeat frequency did not dip below 100 cycles s^−1^ for more than 2 s during the experiment; 17 out of 19 flies prepared were used for analysis. The first 2 s of each trial were discarded while flies adjusted to the new random condition.

**Figure 2.**
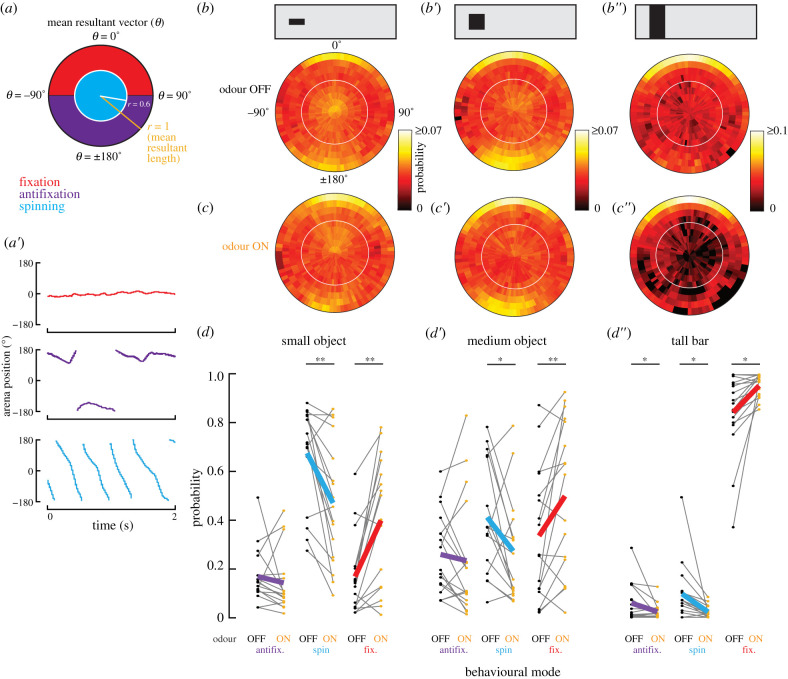
(*a*) Notation of direction (*θ*, degrees), and length (*r*, unitless) of the mean resultant vector of object location. *θ* and *r* values define three behavioural modes. (*a*′) Sample traces of each behavioural mode from a single fly with the 7.5° × 30° object in odourless air. (*b,c*) Mean density of *θ* and *r* in polar coordinates for *n* = 17 flies. White circle indicates *r* = 0.6. (*d*–*d″*) Within-subjects comparison for each behavioural mode. Grey thin lines are for individual flies; coloured thick lines are means for all flies (*n* = 17) (*p < 0.05, **p < 0.01, Student's paired *t*-test).

We calculated probability distributions (figure 1*c*) of the residence time at each azimuthal position for each visual object. Object position traces were averaged in 1 pixel bins (1 pixel = 3.75° azimuth), and averaged across flies (*n* = 17). We plotted azimuthal probability density in polar coordinates (figure 2*b,c*) using a sliding 2 s window analysis to compute mean resultant vector (*θ*), a measure of angular heading in the arena (figure 2*a*), and resultant vector length (*r*), a measure of circular spread of the heading values (figure 2*a*) [12]. Values of *r*, radii along the unit circle, ranged between 0 and 1, with values closer to 1 indicating a narrower spread of unit vectors, or tighter visual control over the visual object, within the window. The probability of each bin of heading values (bin width = 3.75°) and *r* (bin width = 0.1) was averaged across trials and flies (*n* = 17). Each binned measurement was classified for its behavioural mode based on *θ* and *r*. Frontal fixation is defined by −90° < *θ* < 90° (front hemifield) and *r* > 0.6 (figure 2*a*, red zone). Antifixation is defined by −90° > *θ* > 90° (rear hemifield) and *r* > 0.6 (figure 2*a*, purple zone). Spinning is defined as any mean *θ* value with *r* ≤ 0.6 (figure 2*a*, cyan zone). Criteria were based on prior results [1]. From these values, we also calculated a preference index (PI = (attraction responses − aversion responses)/total responses). PI ranged from −1 to 1, with positive values denoting attraction tendency, and negative values denoting aversion tendency (data not shown).

## 3. Results

We assessed how appetitive food odour (apple cider vinegar) influences flies' spatial control over three visual stimuli by computing residence probability of the visual object across flight arena azimuth under closed-loop feedback conditions. A clear peak in residence probability at midline was observed for all three visual objects in clean air, with peak probability proportional to object size (figure 1*c*,*c*′,*c″*; black traces). Conversely, the residence probability of objects within the visual periphery was larger for the small objects than the tall bar. After switching from clean air to odour and repeating the randomized object size trials, the probability of midline object positioning increased for all three visual objects (figure 1*c*,*c*′,*c″*; orange traces, *p < 0.05, Student's paired *t*-test), accompanied by decreased probability at the visual periphery. The effect of odour was most pronounced for the small object (figure 1*c*).

We next calculated the direction (*θ*) and length (*r*) of the mean resultant vector for flies' control of each visual object. We defined frontal fixation as *θ* values in the front hemifield at *r* > 0.6 (figure 2*a*, red region). Antifixation was defined as *θ* in the rear hemifield at *r* > 0.6 (figure 2*a*, purple region). Spinning was defined by *r* ≤ 0.6 (figure 2*a*, cyan region). Data from a single fly highlight instances of all three behavioural modes (figure 2*a*′).

As with the residence probability distributions (figure 1*c*), increasing object size in clean air results in progressively stronger frontal fixation (higher probability values at the circumference near *θ* ≈ 0°), reduced antifixation (higher probability values at the circumference near *θ* ≈ 180°), and reduced spinning (lower probability values near the origin) (figure 2*b*). By visual inspection, for all three visual stimuli, switching from clean air to odour was accompanied by an increase in frontal fixation that is offset by a decrease in spinning (figure 2*c*). Accordingly, the PI increased significantly with the transition from odour OFF to ON for all three visual objects (*p* < 0.01, Student's paired *t*-test, data are redundant with results of figure 1*c* and thus not shown).

We next computed the probability that flies engage in each behavioural mode under each experimental condition. In general, the frequency of antifixation or spinning decreases in the presence of odour for all three visual objects (figure 2*d*, *d*′,*d*″, purple and cyan). Conversely, odour increased frontal fixation behaviour for all visual stimuli (figure 2*d*,*d*′,*d*″; *p < 0.05, **p < 0.01, Student's paired *t*-test). Here, we show the effects for each fly (thin grey lines), and for each experimental condition, in which odour and clean air trials were interspersed. The effects of odour on visual behavioural modes were similar for the very first odour trial as well, suggesting that the influence of odour was immediate and not experience-dependent (data not shown).

## 4. Discussion

Rigidly tethered flies tend to steer syn-directionally in response to an object moving across the visual midline. Thus, under virtual closed-loop feedback conditions, the object becomes fixated near the visual midline [1,2]. Smaller objects are frontally fixated less robustly (figure 1*c*,*c*′,*c″*). In the presence of odour, flies more strongly fixate any size of visual object, while concomitantly decreasing antifixation and spinning (figures 1*c* and 2*b–d*). The effects of odour on both the distribution of behavioural modes and increased fixation would combine to bring a fly closer to a visual object, a behavioural response that has been observed in flies freely exploring a wind tunnel [6]. The modulation of visual salience by an appetitive odour can enhance foraging performance when meaningful sensory signals converge, and conserve neural processing resources when they do not.

Tethered flight experiments are crucial for exploring mechanistic interactions between sensory modalities, since stimuli can be precisely controlled. In open-loop conditions, in which the object is restricted to the visual periphery, flies tend to tonically steer in the opposite direction [1], or execute saccades oriented away from the object [4]. Attractive odours reverse aversion to approach [2]. But why do flies tend to approach (fixate) visual objects under closed-loop feedback conditions (figures 1*c* and 2*b–d*) but avoid them under open-loop conditions? This apparent paradox is resolved by the fact that the valence of a visual stimulus can vary across the visual azimuth. For example, a narrow grating or bar oscillating across midline elicits syn-directional steering responses [13]. Intuitively, this reaction would lead to frontal fixation under closed-loop conditions. Indeed, a model of directionally selective motion detectors flanking the visual midline is sufficient to explain frontal bar fixation [14]. However, positioning a bar or grating in the visual periphery generates a tonic steering effort and wing saccades oriented away from the grating [4,10,15,16]. Thus, the same visual cue triggers different behavioural outcomes depending on its location in the visual field [17]. Under tethered closed-loop control conditions, a visual object stimulates the entire visual azimuth, thereby driving motor responses with different azimuthal tuning.

We do not know whether fixation, antifixation or spinning behaviours are coordinated by different neural pathways. If so, then each subsystem may be individually and differentially modulated by odour. Alternatively, odour modulation may occur after signals from each subsystem have converged upon premotor descending neurons. Our behavioural results provide a conceptual framework for studying these interactions at the neuronal circuit level.

## Data accessibility

Data and Matlab plotting code are available at: https://datadryad.org/stash/dataset/doi:10.5068/D1GD5F [18]. Add the directory to the Matlab path, and run *.PublicAnalysis.m.
